# Thalamic Activity Regulates Interneuron Density in the Developing Visual Thalamus

**DOI:** 10.1523/JNEUROSCI.0247-25.2026

**Published:** 2026-05-19

**Authors:** Irene Huerga-Gómez, Daniel Torres-Romero, Pablo Castellano-Ruiz, Emily Sarah Wilson, Francisco J. Martini, Guillermina López-Bendito

**Affiliations:** Instituto de Neurociencias de Alicante, Universidad Miguel Hernández-Consejo Superior de Investigaciones Científicas (UMH-CSIC), San Juan de Alicante, Alicante 03550, Spain

**Keywords:** GABAergic interneurons, intrinsic activity, mice, thalamus, visual cortex

## Abstract

Neural activity is a fundamental driver of early circuit assembly, yet how it shapes the distribution of inhibitory neurons across sensory networks remains poorly understood. Establishing an appropriate balance between excitation and inhibition is essential for effective sensory processing, but the contribution of activity-dependent mechanisms to interneuron allocation across subcortical and cortical stations is unclear. Here, we use region-specific transgenic mouse models of either sex to selectively manipulate activity at distinct loci and developmental stages of the visual pathway. We show that intrinsic thalamic activity is a key regulator of interneuron density in the dorsolateral geniculate nucleus during early postnatal development. Disruption of thalamic activity leads to persistent increases in interneuron proportion, independent of retinal axon targeting. Moreover, altered thalamic activity propagates to the cortex, producing layer-specific changes in parvalbumin- and somatostatin-expressing interneuron populations in primary visual cortex. Together, our findings identify intrinsic thalamic activity as a central organizer of inhibitory circuit assembly across the visual system, coordinating interneuron integration in both thalamus and cortex during critical developmental windows.

## Significance Statement

The proper integration of inhibitory interneurons into thalamic circuits is essential for establishing balanced excitatory–inhibitory activity in the visual system. Previous work has highlighted the role of retinal input in this process, but the contribution of intrinsic thalamic activity has remained unclear. Here, we show that thalamic activity is critical for determining interneuron density in the dorsolateral geniculate nucleus. In contrast, removal of retinal input alters interneuron positioning but not overall density. These findings reveal that intrinsic thalamic activity and retinal input act through distinct yet complementary mechanisms to shape thalamic inhibitory circuits, with lasting consequences for cortical circuit maturation.

## Introduction

Interneurons comprise a highly heterogeneous neuronal population distinguished by diverse morphologies, electrophysiological properties, and molecular identities. This diversity enables them to regulate the firing patterns of principal neurons and maintain the balance between excitation and inhibition (E/I) within neural circuits (DeFelipe, 1997; Markram et al., 2004; Williams and Riedemann, 2021; Kessaris and Denaxa, 2023). Proper maturation and integration of INs into developing circuits are essential for normal brain function and are strongly influenced by neuronal activity and sensory experience (Cobos et al., 2005; De Marco García et al., 2011, 2015; Anastasiades et al., 2016; Tuncdemir et al., 2016; Duan et al., 2020; Modol et al., 2020). However, the precise contributions of intrinsic thalamic activity to interneuron integration remains poorly understood.

Neuronal activity regulates migration, morphological maturation, and subtype-specific integration of cortical interneurons (De Marco García et al., 2011). In contrast, the mechanisms governing interneuron integration into the thalamus are far less defined. Local thalamic GABAergic interneurons, which are critical modulators of sensory processing, originate outside the thalamus in the midbrain (Jager et al., 2016, 2021) and migrate into sensory nuclei, particularly the visual first-order dorsolateral geniculate (dLG) nucleus, during late embryogenesis. Their numbers increase during early postnatal development, and their spatial distribution is subsequently influenced by retinal axons and Stage II retinal waves (Golding et al., 2014; Charalambakis et al., 2019; Su et al., 2020; Somaiya et al., 2022). Because these interneurons fine-tune thalamic relay activity and visual signal processing (Sillito and Kemp, 1983; Holdefer et al., 1989; Wang et al., 2007, 2011; Morgan and Lichtman, 2020), disruptions in their integration can profoundly alter visual circuit assembly (Jager et al., 2016, 2021). Yet, whether intrinsic thalamic activity contributes to their recruitment and positioning remains largely unexplored.

Recent studies emphasize the thalamus as critical in shaping sensory cortical areas, even prior to the onset of sensory experience (Herrmann and Shatz, 1995; Hanganu et al., 2002; Uesaka et al., 2007; Zhao et al., 2009; Barkat et al., 2011; Mire et al., 2012; Viswanathan et al., 2012; Castillo-Paterna et al., 2015; Moreno-Juan et al., 2017; Antón-Bolaños et al., 2018; Molnár et al., 2020). During embryogenesis, thalamic neurons generate intrinsic activity independently of peripheral input. By embryonic day 14 (E14) in mice, this intrinsic activity emerges as coordinated calcium waves, initially restricted to first-order nuclei and later propagating to higher-order nuclei (Moreno-Juan et al., 2017; Martini et al., 2021). These large-scale synchronous events decline around birth, giving way to smaller and less synchronized postnatal activity patterns. As development proceeds, sensory-driven input refines these activity patterns, contributing to the maturation and stabilization of thalamocortical circuits (Martini et al., 2021).

Here, we investigated how developmental perturbations of neuronal activity influence the integration of local thalamic interneurons into the dLG and the organization of cortical interneurons in primary visual cortex (V1). We employed complementary region-specific mouse models to dissociate the contributions of retinal and thalamic activity. First, we used the *SertCre^Kir^* model, in which activity is perturbed in both ipsilateral retinal ganglion cells (García-Frigola and Herrera, 2010) and in thalamic relay neurons (Chen et al., 2015) under the control of the serotonin transporter. To isolate retinal contributions, we blocked Stage I retinal waves or performed embryonic bilateral enucleation (embBE). To selectively manipulate intrinsic thalamic activity while preserving retinal projections, we used the *Th^Kir^* model (Antón-Bolaños et al., 2019). Our results show that intrinsic thalamic activity regulates interneuron density in the visual thalamus and shapes inhibitory circuits in downstream cortex. Retinal input refines the spatial distribution of thalamic interneurons, whereas disrupted thalamic activity changes interneuron proportion in the dLG and alters the laminar distribution of somatostatin (SST) and parvalbumin (PV) interneurons in V1. Together, these findings reveal a coordinated, temporally structured interaction between retinal input and intrinsic thalamic activity that guides inhibitory circuit assembly across thalamic and cortical networks during development.

## Materials and Methods

### Mouse strains

All animal procedures were approved by the Committee on Animal Research at the Universidad Miguel Hernández de Elche and in compliance with the Spanish and European Union regulations.

Transgenic lines were maintained on an ICR/CD-1 background and genotyped by PCR. The gestational age of plug formation (E0.5) was assumed for experimental consistency. For *SertCre^Kir^* animals, R26Kir2.1-mCherry floxed mice were crossbred with the SertCre+ mouse line (JAX:014554; Zhuang et al., 2005). In this line, Kir2.1 expression is driven by Cre under the serotonin transporter (Sert) promoter, resulting in gradual and tamoxifen-independent onset of Kir2.1 expression within retinal ganglion cells and diverse thalamic nuclei from late gestation through early postnatal stages. The R26tdTomato Cre-dependent mouse strain (stock number 007908) was procured from Jackson Laboratories. The GAD67-GFP mouse model, characterized by the expression of the green fluorescent protein (GFP) under the interneuron-specific GAD67 promoter, was employed to label the interneuron populations within the central nervous system (CNS; Tamamaki et al., 2003).

The R26Kir2.1-mCherry floxed mice were crossbred with the Gbx2CreERT/+ mouse line, in which CreERT2 expression is driven by the thalamus-specific Gbx2 promoter. The resultant mutant, herein referred to as *Th^Kir^*, were generated via tamoxifen administration (5 mg dissolved in corn oil, administered via gavage) at E10.5, enabling selective labeling of primary sensory nuclei within the thalamus (Moreno-Juan et al., 2017; Antón-Bolaños et al., 2019). The strain GAD67-GFP:Gbx2-Kir2.1 was derived from the Gbx2-Kir2.1 line. To mitigate potential effects of early birth resultant from tamoxifen exposure, intraperitoneal administration of progesterone (DEPO-PROGEVERA, 125 mg/kg) was conducted at E14.5. C-sections were performed on pregnant females at E19.5, with resultant pups fostered by surrogate mothers. CreERT2-negative littermates served as control subjects across all experimental procedures.

### In utero bilateral enucleation

Embryonic bilateral enucleation was performed on pregnant females at E14.5, which were deeply anesthetized with isoflurane as previously described (Herrmann and Shatz, 1995; Moreno-Juan et al., 2017, 2023). The uterine horns were exposed after a midline laparotomy. Both eyes were cauterized in half of the litter, and then the embryos were placed back in the abdominal cavity. The surgical incision was closed, and the embryos developed normally until birth date and postnatal stages.

### Immunohistochemistry

Mice were perfused with paraformaldehyde (PFA) 4% in PBS 0.01 M. The brains were dissected and postfixed overnight. In contrast, brains at embryonic stages were directly dissected and fixed in 4% PFA overnight. Coronal sections 60 µm thick were obtained with the vibratome. Brain slices were then treated with a citrate buffer at pH 6 to unmask the antigens. Then, slices were washed and the blocking solution containing 10% normal goat serum (NGS) and 0.3% Triton X-100 (Tx100) was placed for 1 h. Slices were incubated overnight at 4°C with the respective primary antibodies, 3% NGS, 0.3% Tx100 in PBS 0.01 M: guinea pig anti-vGlut2 (1:5,000, Synaptic Systems, #135404), chicken anti-GFP (1:2,000, Aves Labs, #GFP-1020), rat anti-RFP (1:1,000, ChromoTek, #5F8), mouse anti-NeuN (1:1,000, Merck Millipore, #MAB377), rabbit anti-Iba1 (1:1,000, Wako #019-19741), mouse anti-Otx2 (1:50, courtesy of Prosziank lab), rabbit anti-PV (1:1,000, Swant, #PV27), rat anti-somatostatin (1:50, Merck, #MAB354), rabbit anti-Caspase-3 (1:150, Abcam, #ab2302). Sections were washed several times with PBS 0.01 M and incubated 2 h at room temperature with secondary antibody, 3% NGS, 0.3% Tx100 in PBS 0.01 M: Alexa488 donkey anti-guinea pig (1:500, Thermo Fisher Scientific, #A11073), Alexa546 donkey anti-guinea pig (1:500, Thermo Fisher Scientific, #A11040), Alexa488 goat anti-chicken (1:500, Thermo Fisher Scientific, #A11039), Alexa594 donkey anti-rat (1:500, Thermo Fisher Scientific, #A21209), Alexa647 donkey anti-rabbit (1:500, Jackson, #711-605-152). Brain slices were rinsed in PBS 0.01 M and then stained with DAPI for 5 min. Finally, they were mounted with Fluoromount.

### Confocal microscopy

Images were taken with an inverted Olympus FV1200 confocal microscope with a 20× and 40× oil-immersion objectives, with a *Z*-depth of 3 µm for thalamic slices and 5 µm for the cortex.

### Image quantification

Images were analyzed using the FIJI (FIJI is just ImageJ) software. The quantification of the cells in the dLG was done with the Cell Counter plugin, selecting a ROI defined by the anatomical boundaries of the dLG at four rostrocaudal levels in each brain.

For cortical interneurons, brains were taken at different postnatal stages. From each brain, three different slices covering three rostrocaudal levels were selected. In each cortical layer, the average of interneurons was done analyzing three ROIs of 100 × 100 µm.

For cell death analysis, Caspase3a-positive cells were quantified in coronal sections and normalized to the area of each dLG. The results were normalized to the mean of the control group at P6, representing the fold-change in Caspase3a-positive cells in the other conditions.

### In vivo intraocular injections

For the pharmacological ablation of type I retinal waves in vivo, two experimental windows were tested (E18.5–P0 and P0–P1). For E18.5–P0, pregnant females at E18.5 were deeply anesthetized with isoflurane as previously described (Herrmann and Shatz, 1995; Moreno-Juan et al., 2017, 2023). The uterine horns were exposed after a midline laparotomy. Half of the embryos in each litter received bilateral intravitreal injections of carbenoxolone (10 mM; Cbx, Merck, C4790) and the other half received saline, using a pulled glass micropipette. Embryos were then returned to the abdominal cavity, the surgical incision was closed, and the embryos developed normally until birth date. At P0, pups received a second bilateral injection (Cbx or saline, according to group assignment). Brains were collected at P2 and P6. For the P0–P1 group, early postnatal pups were anesthetized using ice and injected bilaterally with Cbx or saline at both P0 and P1, using a pulled glass micropipette through a small corneal incision. Each eye received 0.3 µl of Cbx or saline per injection. Pups were recovered on a heating pad after each procedure, and brains were collected at P2 and P6.

### Ex vivo calcium imaging

Brains from postnatal mice (P0 and P4) were rapidly removed and transferred to ice-cold, carbogenated slicing solution containing the following (in mM): 2.5 KCl, 7 MgSO_4_, 0.5 CaCl_2_, 1 NaH_2_PO_4_, 26 Na_2_HCO_3_, 11 glucose, and 228 sucrose. Coronal 300 µm slices were cut on a vibratome (VT1200, Leica). Slices recovered for 30 min at room temperature in standard ACSF containing the following (in mM): 119 NaCl, 5 KCl, 1.3 MgSO_4_, 2.4 CaCl_2_, 1 NaH_2_PO_4_, 26 Na_2_HCO_3_, and 11 glucose. All solutions were continuously bubbled with 95% O_2_ and 5% CO_2_ carbogen. Slices were loaded with Cal520 (AAT Bioquest) by incubation for 45 min at 37°C and then washed to remove excess dye and transferred to the imaging chamber of a Leica Thunder microscope, where slices were incubated for 30 min prior to recording. The Thunder chamber was maintained at 32°C and supplied with carbogen throughout imaging.

Imaging was performed using a 10× objective. Frames were acquired with a 100 ms exposure and a 300 ms interframe interval (corresponding to ∼3.33 Hz acquisition rate). For each animal, one slice was recorded for a single 15 min epoch. Calcium imaging data were analyzed as described previously (Moreno-Juan et al., 2017; Antón-Bolaños et al., 2019). Briefly, the dLG was delineated in each slice and this area was divided into square ROIs. Each ROI approximated the size of a single cell. Calcium events were detected in each ROI and the fraction of the total ROIs active and the frequency of events per ROI were quantified throughout the movie.

### Statistics

Statistical analysis was performed using SPSS and R. The statistical comparison between two specific populations was done using a two-tailed Student’s *t* test with Welch correction (not assuming equal variances). Mann–Whitney tests for nonparametric data were applied to data which failed the Kolmogorov–Smirnov normality test.

Analyses of interneuron density, number, area, and dorsoventral distribution in the dLG and cortex were performed using generalized linear mixed models (GLMMs). To facilitate interpretation, datasets were divided into independent subsets according to genotype/intervention (*SertCre^Kir^*, *embBE*, *Th^Kir^*) and, for cortical data, also by interneuron type. Separate models were fitted to each subset. For the cortex, data from different ROIs and rostrocaudal sections were aggregated by summation, since these variables did not contribute significantly to the variance.

Density of interneurons was modeled with a Tweedie distribution and a log link. dLG section area was modeled identically. The number of interneurons was modeled with a negative binomial distribution (*nbinom1* parametrization, variance proportional to the mean) and a log link. Dorsoventral distribution was modeled with a binomial distribution and a logit link, using paired counts of interneurons across dorsoventral bins as the response. For the dLG, fixed effects included rostrocaudal section, age, and genotype/intervention (with interactions). For the cortex, fixed effects included cortical layer, age, and genotype/intervention (with interactions). In all cases, animal identity was included as a random intercept.

Models were fitted by maximum likelihood (ML) using the glmmTMB package in R (Brooks et al., 2017). Post hoc tests for simple effects of genotype/intervention, age, and rostrocaudal section or layer were conducted with the emmeans package (Lenth and Piaskowski, 2026), applying Holm correction for multiple comparisons where appropriate. *p* values <0.05 were considered statistically significant and are indicated as follows: *p < 0.05; **p < 0.01; ***p < 0.001. The sample sizes are reported in the figure legends. *p* values of boxplots representing the average density across the four rostrocaudal levels are also indicated in the figure legends. Significant *p* values for individual rostrocaudal sections are listed below.

**Figure 1.**
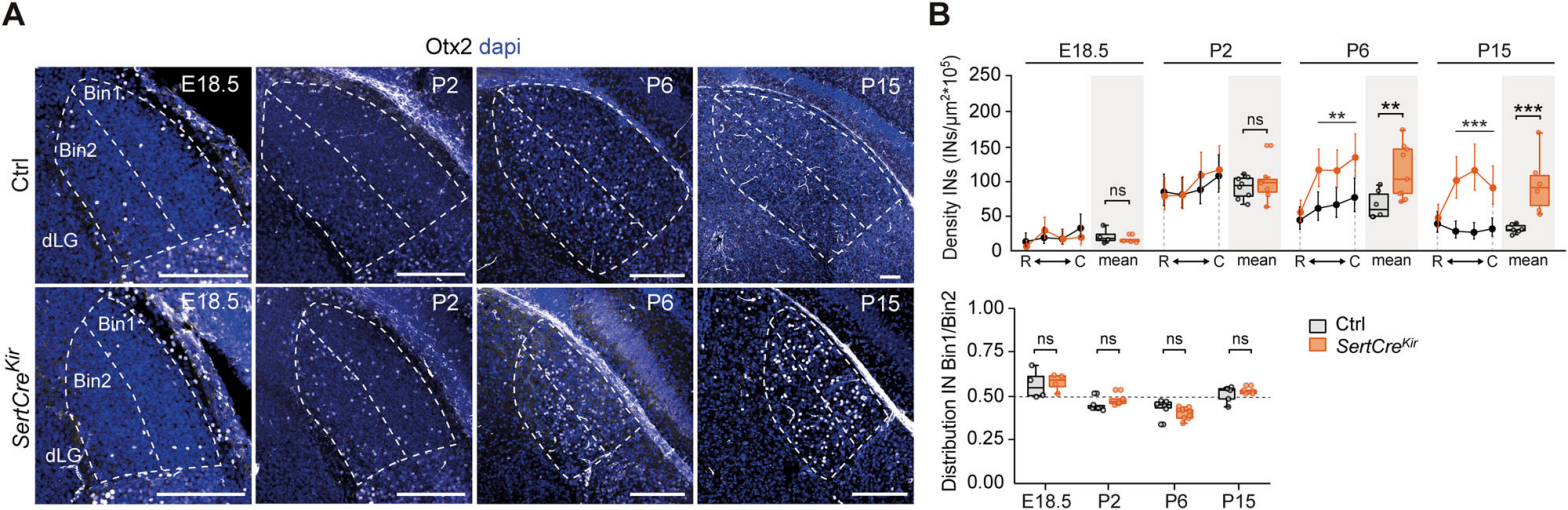
Activity-dependent modulation of dLG interneuron density. ***A***, Representative coronal sections illustrating the distribution of thalamic interneurons (INs) at different developmental time points in control and *SertCre^Kir^* littermates. ***B***, Quantification of interneuron density across four rostrocaudal (R–C) levels of the dLG in control and *SertCre^Kir^* animals at different ages. Mean ± 95% confidence intervals are shown for each R–C level. Boxplots represent the average density across the four R–C levels per animal. Bottom, Proportion of interneurons in the dorsal bin (bin1), calculated as cells in bin1 / (cells in bin1 + cells in bin2). Data were analyzed using a GLM with gamma distribution (see Materials and Methods). Density comparisons: E18.5 *n* = 4, n.s., P2 *n* = 8, n.s., P6 *n* = 6–9, *p* = 0.0031, P15 *n* = 6–8, *p* < 0.001. Dorsoventral distribution: E18.5 *n* = 6–7, n.s; P2 *n* = 6, n.s, P6 *n* = 8–9, n.s, P15 *n* = 12–14, n.s. Scale bars, 100 µm. IN, interneuron.

In *SertCre^Kir^*, for Figure 1*B*, at P6: section 2 *p* = 0.0016; section 3 *p* = 0.0060; section 4 *p* = 0.0040. At P15: sections 2–4 *p* < 0.001. For Figure 1*C*, at P15: section 1 *p* = 0.0044. For Figure 1*D*, at P2: section 3 *p* = 0.0449; at P6: section 1 *p* = 0.0116: sections 2–4 *p* < 0.001; at P15: sections 1–4 *p* < 0.001.

**Figure 2.**
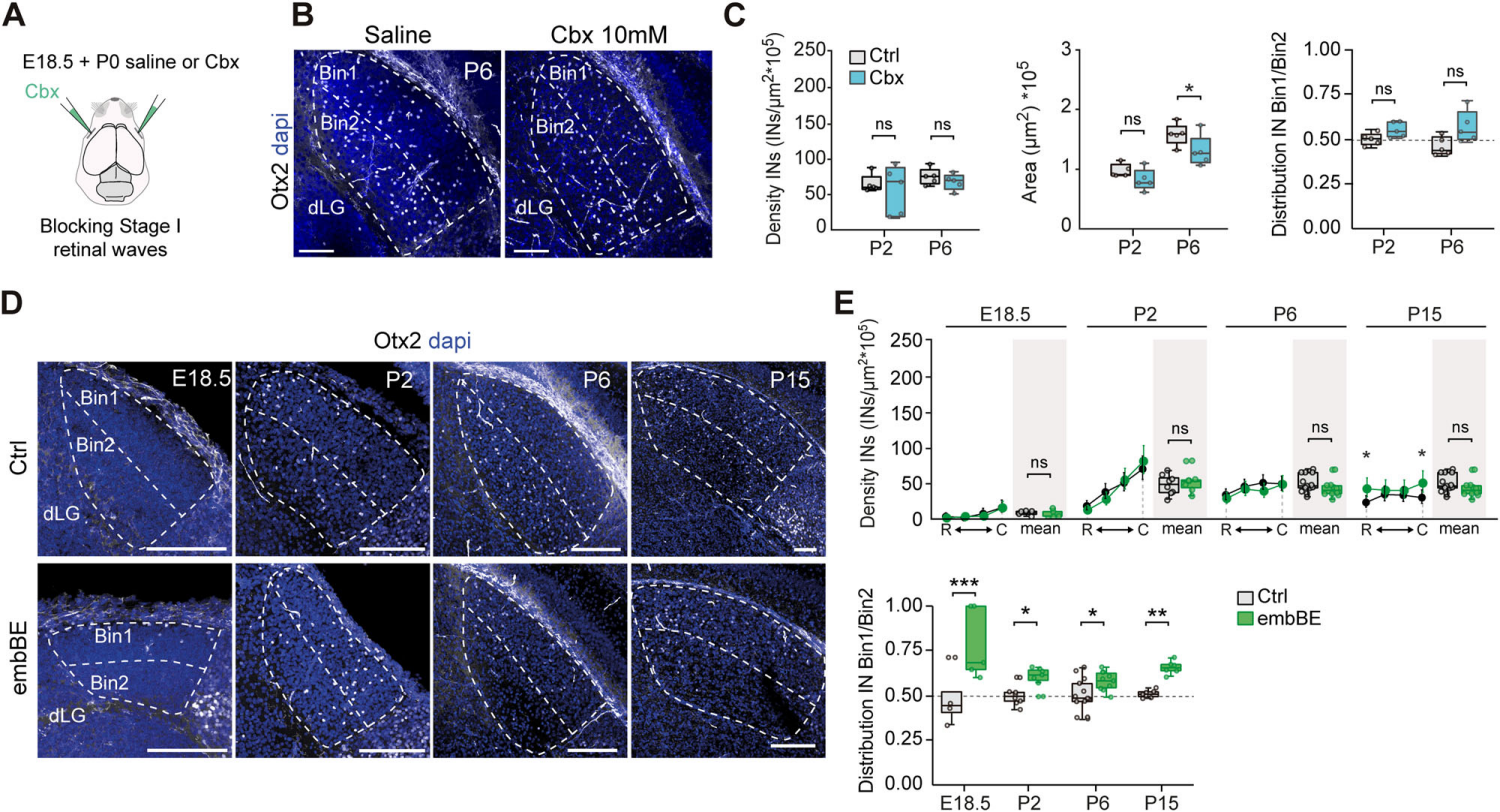
Interneuron integration into the dLG depends on retino-thalamic input but not on Stage I retinal waves. ***A***, Schematic representation of the experimental manipulations performed at E18.5 and P0. ***B***, Representative coronal sections showing the distribution of thalamic interneurons (INs) at P6 in control and carbenoxolone (Cbx)-injected mice. ***C***, Quantification of interneuron density, dLG area, and dorsoventral distribution (Bin1/Bin2) in control and Cbx-injected animals at P2 and P6. P2 *n* = 5 n.s., P6 *n* = 5 n.s. ***D***, Representative coronal sections showing the distribution of thalamic interneurons at different developmental time points in control and embryonic bilateral enucleation (embBE) littermates. ***E***, Quantification of interneuron density across four rostrocaudal (R–C) levels of the dLG in control and embBE animals at different ages. Mean ± 95% confidence intervals are shown for each R–C level. Boxplots represent the average density across the four R–C levels per animal. Bottom, Proportion of interneurons in the dorsal bin (bin1), calculated as cells in bin1 / (cells in bin1 + cells in bin2). Data were analyzed using a GLM with Gamma distribution (see Materials and Methods). Density comparisons: E18.5 *n* = 5 n.s.; P2 *n* = 8 *n.s*, P6 *n* = 11–13 n.s., P15 *n* = 6–7 n.s.; dorsoventral distribution: E18.5 *n* = 5 *p* < 0.001; P2 *n* = 8 *p* *=* 0.0181, P6 *n* = 11–13 *p* *=* 0.0144, P15 *n* = 6–7 *p* *=* 0.0042. Scale bar, 100 µm. IN, interneuron.

In embBE, for Figure 2*E*, at P15: section 1 *p* = 0.0101; section 4 *p* = 0.0175. For Figure S2*D*, at P2: section 2 *p* = 0.0016. At P6: section 1 *p* = 0.001; sections 2–4 *p* < 0.001. At P15: section 2 *p* = 0.0137; sections 3 *p* = 0.0087. For Figure S2*E*, at P2: section 1 *p* = 0.0118; section 2 *p* < 0.001; section 2 *p* < 0.001; section 3 *p* = 0.0163. At P6: sections 1–4 *p* < 0.001. At P15: sections 1–4 *p* < 0.001.

**Figure 3.**
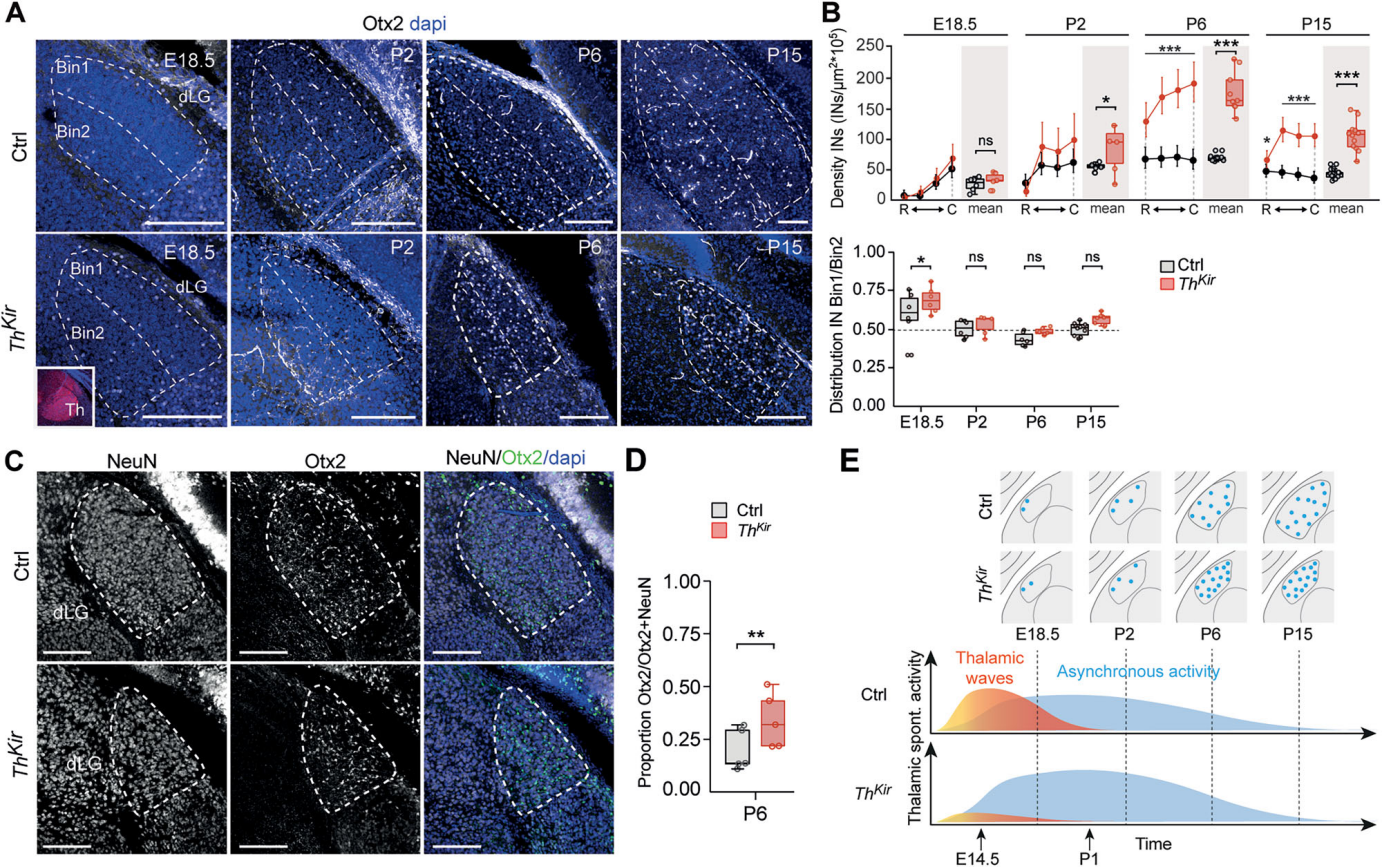
Altered thalamic intrinsic activity affects dLG interneuron density and distribution. ***A***, Representative coronal sections showing the distribution of thalamic interneurons (INs) at different developmental stages in control and *Th^Kir^* mice. The dLG was subdivided into two bins: Bin 1 (upper tiers, dorsal half of the dLG) and Bin 2 (lower tiers, ventral half). Inset, Kir2.1 expression in the dLG. ***B***, Quantification of interneuron density across four rostrocaudal (R–C) levels in control and *Th^Kir^* animals at different ages. Mean ± 95% confidence intervals are shown for each R–C level. Boxplots represent the average density across the four R–C levels per animal. Data were analyzed using a generalized linear model (GLM) with gamma distribution (see Materials and Methods). Bottom, Proportion of interneurons in the dorsal bin (bin1), calculated as cells in bin1 / (cells in bin1 + cells in bin2). Density comparisons: E18.5 *n* = 6–7, n.s.; P2 *n* = 6, *p* = 0.03; P6 *n* = 8–9, *p* < 0.0001; P15 *n* = 12–14, *p* < 0.0001. Dorsoventral distribution: E18.5 *n* = 6–7, *p* = 0.0339; P2 *n* = 6, n.s.; P6 *n* = 8–9, n.s.; P15 *n* = 12–14, n.s. ***C***, Immunostaining for NeuN and Otx2 at P6. ***D***, Quantification of the proportion of Otx2-positive interneurons relative to total neurons, calculated as Otx2 / (Otx2 + NeuN). P6 (*n* = 5); *p* < 0.01 (two-way ANOVA). ***E***, Schematic summarizing the developmental emergence of thalamic intrinsic activity and the migration dynamics of control and *Th^Kir^* interneurons into the dLG over time. Scale bars, 100 µm. IN, interneuron.

In *Th^Kir^*, for Figure 3*B*, at P6: sections 1–4 *p* < 0. 001. At P15: section 1 *p* = 0.0353; sections 2–4 *p* < 0.001. For Figure S3*A*, at P6: section 2 *p* = 0.0057; section 3 *p* = 0.0166; section 4 *p* < 0.001. At P15: section 1 *p* < 0.001. For Figure S3*B*, at P2: section 2 *p* = 0.0375; section 3 *p* = 0.0485. At P6: sections 1–4 *p* < 0.001. At P15: sections 1–4 *p* < 0.001.

The proportion of active ROIs was modeled via a binomial GLM with a logit link function, utilizing the counts of active and silent ROIs. Genotype/intervention was defined as a fixed factor. Event frequency was modeled using a negative binomial GLMM with a log link function, with genotype/intervention, age and their interactions as fixed factors, and with animal identity as a random factor to address pseudoreplication from multiple ROIs per animal.

For Figure S1*F* at P4, *p* = 0.0022, and for Figure S1*G* at P4 *p* = 0.0211.

## Results

### Interneuron integration into the developing visual thalamus is activity-dependent

Interneurons destined for the thalamus are generated between E10 and E13 and begin invading the caudal nascent thalamus from E18.5 until the end of the first postnatal week. This process is partially guided by retinal input, as genetic and pharmacological models that disrupt retinal activity or retinal projections display impaired interneuron migration, as well as abnormal morphology and synaptic connectivity (Golding et al., 2014; Charalambakis et al., 2019). Based on these findings, we hypothesized that intrinsic thalamic activity may also contribute to interneuron integration within visual circuits.

To directly test how neuronal activity influences interneurons integration into the dorsolateral geniculate (dLG) nucleus, we used the *SertCre^Kir^* (also known as Slc6a4-Kir2.1) mouse line, which exhibits disrupted intrinsic activity in both the retina and thalamus. In this model, mice show a progressive increase in Kir2.1 expression starting at E18 in the ventral posterior nucleus (VPM), followed by gradual expansion to encompass sensory thalamic nuclei, including the dLG, during early postnatal development (Fig. S1*A–C*). In the retina, Kir2.1 overexpression disrupts retinal ganglion cell excitability (Negueruela et al., 2024), thereby altering the normal network activity. Ex vivo calcium imaging of the early postnatal thalamus revealed that, although coordinated waves of activity were virtually absent in the dLG in both control and *SertCre^Kir^* mice, overall thalamic activity was markedly disrupted in *SertCre^Kir^* mutants. Recordings at P4 showed a pronounced increase in asynchronous activity within the dLG of *SertCre^Kir^* mice compared with controls, a trend that was already detectable at P0, consistent with the onset of Sert expression in this nucleus (Fig. S1*D–G*). An increase in asynchronous network activity following Kir2.1 overexpression has previously been reported in immature thalamic neurons (Antón-Bolaños et al., 2019). Therefore, because *SertCre^Kir^* mice exhibit altered activity in both retinal and thalamic compartments, this model provides a powerful system to investigate how broadly perturbed perinatal retino-thalamic activity impacts interneuron integration into developing visual thalamic circuits.

To determine how interneuron integration is affected across this altered activity landscape, we quantified the density of interneurons expressing Otx2, a key transcription factor required for thalamic interneuron development (Golding et al., 2014). Analyses were performed at four rostrocaudal levels of the dLG across multiple developmental stages (E18.5, P2, P6, and P15) in *SertCre^Kir^* mice and control littermates (Fig. 1*A*). Disruption of activity in *SertCre^Kir^* mice resulted in a significant increase in Otx2+ interneuron density at P6 and P15 (Fig. 1*B*). Importantly, the total number of interneurons was comparable between *SertCre^Kir^* mice and controls (Fig. S1*H*), indicating that the observed increase in density does not reflect enhanced interneuron production or survival. Instead, *SertCre^Kir^* mice exhibited a failure of normal postnatal dLG growth, consistent with reduced dLG size following Kir2.1 overexpression (Fig. S1*I*). Moreover, interneurons maintained a broad dorsoventral distribution similar to that observed in control littermates (Fig. 1*B*), suggesting that large-scale spatial allocation within the nucleus is preserved. Together, these data indicate that perturbation of perinatal retino-thalamic activity alters interneuron density. Since the *SertCre^Kir^* model disrupts activity in both the thalamus and the retina, we next sought to dissect the specific contribution of retinal activity by asking whether early retinal activity alone is sufficient to influence interneuron integration in the developing dLG.

### Retinal input does not influence interneuron density in the dLG

During development, the retina generates spontaneous waves of activity that propagate throughout the visual pathway, including the dLG and visual cortex. In mice, three sequential stages of retinal waves have been defined: Stage I (approximately E16 to P1), Stage II (P1 to P10), and Stage III (P10 until eye opening at approximately P14). Stage II retinal waves have been shown to be essential for proper interneuron distribution, as daily epibatidine treatment from P0 to P10 disrupts their spatial allocation within the dLG (Golding et al., 2014). We therefore asked whether Stage I retinal waves, which occur during the initial phase of interneuron migration into the thalamus, are sufficient to influence interneuron density in the dLG.

To selectively block Stage I retinal waves, we administered carbenoxolone (Cbx), a gap junction inhibitor that disrupts Stage I activity without affecting overall eye development (Guillamón-Vivancos et al., 2022). Cbx (10 mM) was injected bilaterally at E18 and P0 (Fig. 2*A*), coinciding with the onset of interneuron invasion into the dLG. This paradigm has been previously validated to effectively suppress Stage I retinal waves and alter early circuit organization (Guillamón-Vivancos et al., 2022). Interneuron density and distribution were then quantified at P2 and P6. No significant differences were detected in interneuron density or dorsoventral distribution between Cbx-treated and control animals (Fig. 2*B*,*C*). Comparable results were obtained when Cbx was administered at P0 and P1, targeting the final phase of Stage I waves (Fig. S2*A–C*). These data indicate that, although Stage II retinal waves are critical for interneuron distribution, Stage I retinal waves are not sufficient to alter interneuron density or large-scale positioning within the dLG.

To further assess the requirement of retinal input, we next examined the impact of eliminating retinal projections altogether. Bilateral embryonic enucleation was performed in wild-type mice at E14.5, prior to the arrival of retinal axons in the dLG. Consistent with previous reports (Golding et al., 2014; Moreno-Juan et al., 2017, 2023), embryonically enucleated (embBE) mice exhibited a reduction in dLG area beginning at P2 that persisted through the first two postnatal weeks (Fig. S2*E*). Interneuron density within the dLG was not significantly altered (Fig. 2*D*,*E*) because both nuclear size and total number of interneurons was proportionally decreased at P6 (Fig. S2*D*), with additional rostrocaudal level-specific differences emerging at P15. Moreover, at the dorsoventral axis, interneurons accumulated preferentially in the upper tiers of the nucleus in embBE mice (Fig. 2*E*), as previously described by Golding et al. (2014). Together, these results confirm that the structural presence of retino-thalamic projections is required for proper interneuron recruitment and spatial allocation. In contrast, early retinal activity mediated by Stage I waves does not appear to be a major determinant of interneuron density in the developing dLG.

### Disrupting thalamic activity from embryonic stages increases interneuron density in the dLG

Previous studies have established that retinal input and Stage II retinal waves regulate interneuron positioning and number in the dLG (Golding et al., 2014; Charalambakis et al., 2019; Su et al., 2020; Somaiya et al., 2022). However, whether intrinsic thalamic activity itself contributes to interneuron integration has remained unclear.

Although the *SertCre^Kir^* model demonstrated that globally disrupting early retino-thalamic activity alters interneuron density, it does not disentangle retinal from thalamic contributions. Because early retinal activity (Stage I waves) was not sufficient to modify interneuron density, we next isolated the role of thalamic activity using the thalamus-specific *Th^Kir^* mouse line. In this model, the inward-rectifying potassium channel Kir2.1 is overexpressed in postmitotic thalamic neurons following tamoxifen induction at E10.5 under the Gbx2 promoter (Moreno-Juan et al., 2017; Antón-Bolaños et al., 2019). This manipulation disrupts intrinsic membrane properties beginning in mid-gestation, shifting coordinated embryonic thalamic calcium waves into asynchronous activity that persists postnatally (Antón-Bolaños et al., 2019; Martini et al., 2021). Importantly, retino-thalamic axon targeting remains intact in *Th^Kir^* mice (Moreno-Juan et al., 2023), allowing selective assessment of thalamic activity.

Since thalamic interneurons invade the dLG between E18.5 and the end of the first postnatal week (Golding et al., 2014; Jager et al., 2016), we hypothesized that embryonic disruption of thalamic activity would alter their integration. We therefore quantified Otx2^+^ interneuron density at E18.5, P2, P6, and P15. *Th^Kir^* mice exhibited a significant increase in interneuron density at P2, P6, and P15 compared with controls (Fig. 3*B*). This increase reflected a marked reduction in dLG area at these stages, together with a transient rise in the absolute number of interneurons at P6 (Fig. S3*A,B*). Notably, dLG size failed to expand between P2 and P15 in *Th^Kir^* mice, indicating impaired postnatal growth. Despite these changes, interneurons remained evenly distributed along the dorsoventral axis (Fig. 3*B*).

To assess the structural E/I balance, we quantified the proportion of Otx2^+^ interneurons relative to total neurons. Although overall cell density (Nissl) was unchanged (Fig. S3*C,D*), *Th^Kir^* mice displayed a significantly increased interneuron proportion at P6 that persisted at P15 (Fig. 3*C*,*D*; Fig. S3*E,F*), indicating a shift in the cellular E/I ratio. While the persistent elevation in density was largely attributable to reduced nuclear size, the transient increase in absolute interneuron number at P6 suggested a potential contribution of altered cell survival. To test this possibility, we crossed *Th^Kir^* mice with GAD67-GFP reporter mice (Tamamaki et al., 2003). GFP strongly colocalized with Otx2 (93% ± 2.2%, SEM), validating its use as an interneuron marker (Fig. S4*A,B*). Although cleaved Caspase-3 staining at P6 revealed increased apoptosis in *Th^Kir^* mice, consistent with previous reports (Moreno-Juan et al., 2023), apoptotic cells did not overlap with GFP^+^ interneurons (Fig. S4*C–F*). Thus, altered interneuron number and proportion are not explained by changes in interneuron cell death. Together, these findings demonstrate that disrupting intrinsic thalamic activity from embryonic stages leads to a transient accumulation of interneurons and a persistent increase in interneuron density in the dLG. These results identify thalamic intrinsic activity as a key regulator of interneuron integration during early postnatal development (Fig. 3*E*).

### Cortical interneuron density is affected by manipulation of thalamic activity

Our previous findings demonstrated that disruption of thalamic activity increases interneuron proportion in the dLG. As thalamic activity influences cortical circuit assembly before the onset of sensory experience (Moreno-Juan et al., 2017; Antón-Bolaños et al., 2019), and *Th^Kir^* mice display enhanced cortical excitability (Antón-Bolaños et al., 2019), we next asked whether embryonic thalamic desynchronization also affects cortical interneuron development.

**Figure 4.**
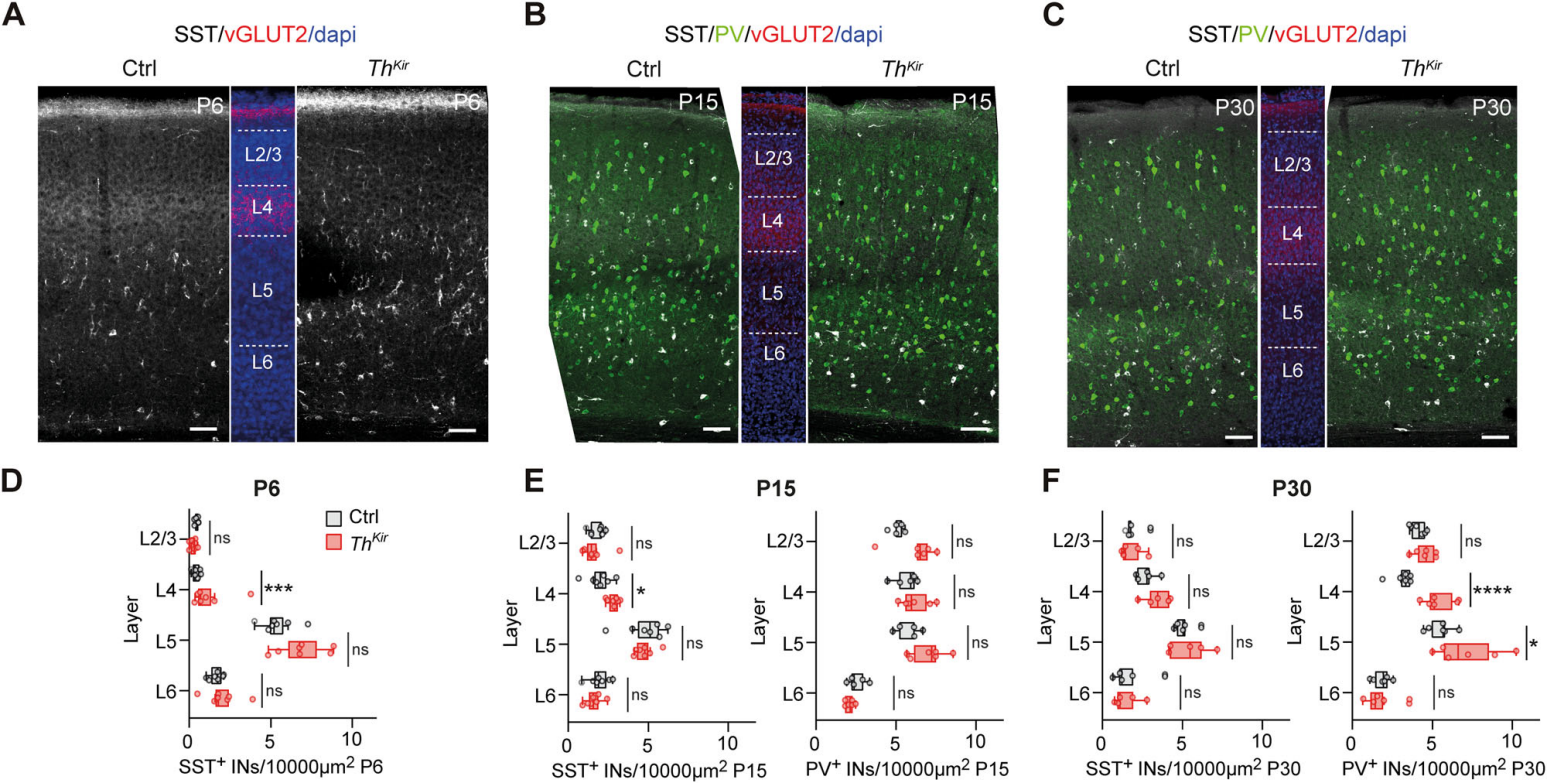
Intrinsic thalamic activity influences cortical interneuron density in V1. ***A–C***, Representative coronal sections of primary visual cortex (V1) showing the laminar distribution of SST^+^ and PV^+^ interneurons at P6, P15, and P30. ***D***, Quantification of SST^+^ interneuron distribution across cortical layers in V1 at P6 in control and *Th^Kir^* littermates. ***E***, ***F***, Quantification of SST^+^ and PV^+^ interneuron distribution across cortical layers at P15 and P30 in control and *Th^Kir^* littermates. All graphs are presented as boxplots of data fitted using a mixed generalized linear model (GLM) with gamma distribution (see Materials and Methods). SST+: P6 *n* = 7 (L4 *p* = 0.0002), P15 *n* = 7 (L4 *p* = 0.0165), P30 *n* = 5–6. PV+: P15 *n* = 5–6, P30 *n* = 6 (L4 *p* = 0.0002; L5 *p* = 0.03). Scale bar, 100 µm. SST, somatostatin; PV, parvalbumin; IN, interneuron.

We first quantified overall interneuron density in primary visual cortex (V1). *Th^Kir^* mice exhibited a transient increase in GAD67^+^ interneuron density in layers 4 (L4) and 5 (L5) at P15, which normalized by P30 (Fig. S5*A–F*). Cortical thickness remained unchanged across layers (Fig. S5*G–I*), arguing against gross anatomical defects. To determine whether specific interneuron subtypes were differentially affected, we examined somatostatin-positive (SST^+^) and parvalbumin-positive (PV^+^) populations at P6, P15, and P30. SST^+^ interneurons, which are detectable early postnatally (Duan et al., 2020; Williams and Riedemann, 2021), were transiently increased in L4 at P6 and P15 in *Th^Kir^* mice, returning to control levels by P30 (Fig. 4). In contrast, PV^+^ interneurons, which mature later (del Río et al., 1994; Meyer et al., 2002), showed no early differences but were significantly increased in L4 and L5 at P30 (Fig. 4*C*,*F*). Together with preserved cortical thickness and the previously reported persistent barrel map defects in *Th^Kir^* mice (Antón-Bolaños et al., 2019), these data indicate that the observed changes do not reflect a generalized developmental delay. Rather, prenatal thalamic activity disruption selectively alters the density and laminar allocation of SST^+^ and PV^+^ interneurons, particularly within L4, the principal thalamo-recipient layer.

**Figure 5.**
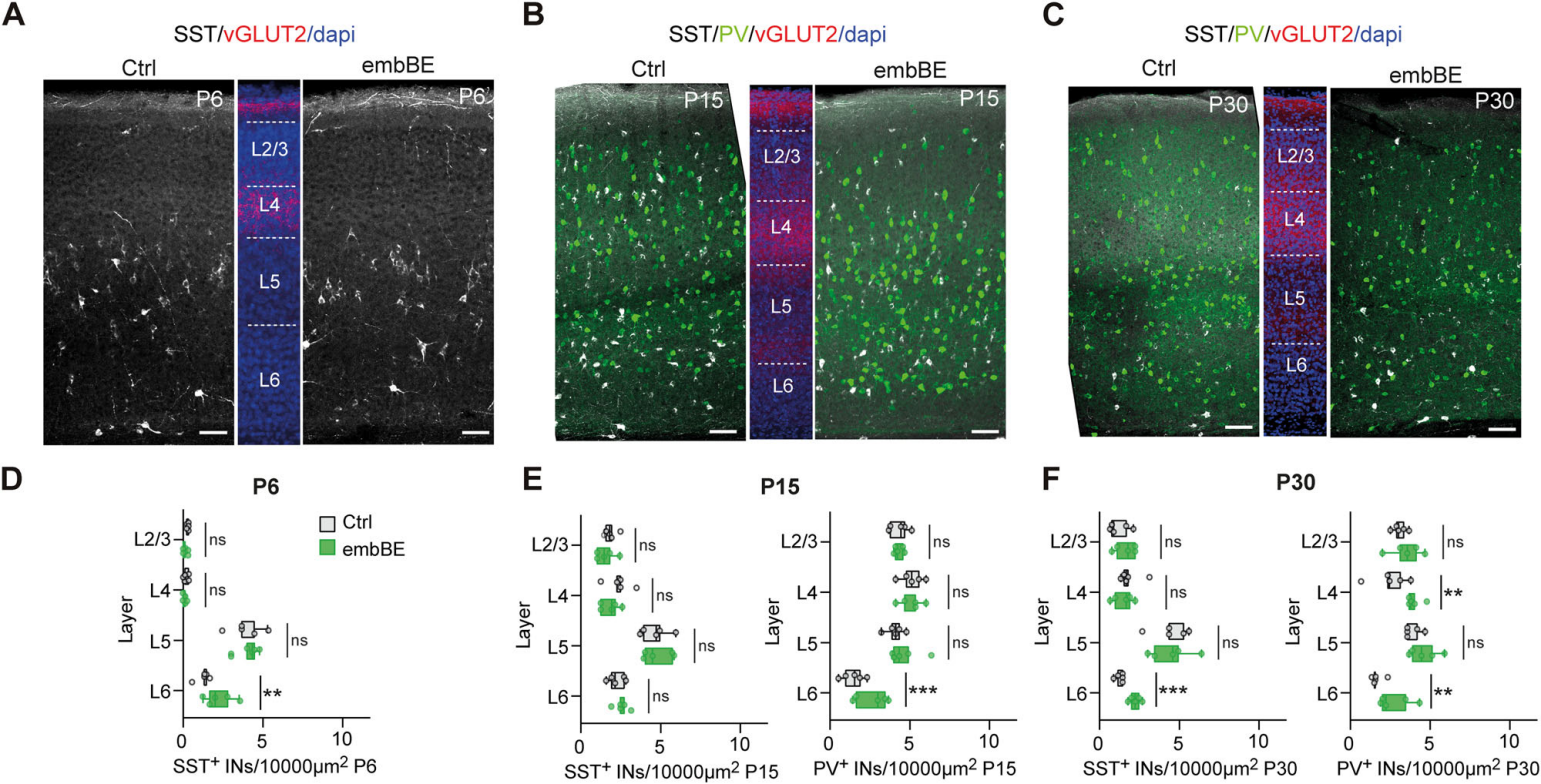
Embryonic bilateral enucleation alters cortical interneuron distribution in V1. ***A–C***, Representative coronal sections of primary visual cortex (V1) showing the laminar distribution of SST^+^ and PV^+^ interneurons at P6, P15, and P30 in control and embryonic bilateral enucleation (embBE) animals. ***D***, Quantification of SST^+^ interneuron distribution across cortical layers in V1 at P6 in control and embBE littermates. ***E***, ***F***, Quantification of SST^+^ and PV^+^ interneuron distribution across cortical layers at P15 and P30 in control and embBE littermates. All graphs are presented as boxplots of data fitted using a mixed generalized linear model (GLM) with gamma distribution (see Materials and Methods). SST+: P6 *n* = 5 (L6 *p* = 0.0011), P15 *n* = 5, P30 *n* = 5 (L6 *p* = 0.0013). PV+: P15 *n* = 5 (L6 *p* = 0.0003), P30 *n* = 5 (L4 *p* = 0.0024; L6 *p* = 0.0024). Scale bar, 100 µm. SST, somatostatin; PV, parvalbumin; IN, interneuron.

To assess the consequences of complete and permanent loss of retinal input, we next analyzed V1 interneurons in embBE mice. SST^+^ interneurons showed a transient increase in layer 6 (L6) at P6, normalized at P15, and re-emerged by P30 (Fig. 5). PV^+^ interneurons displayed a similar laminar bias, with increases in L4 and L6 that became evident at later stages (Fig. 5*E*,*F*). Collectively, these findings demonstrate that subcortical perturbations exert layer-specific effects on cortical interneuron organization. Disruption of intrinsic thalamic activity preferentially affects interneurons in thalamo-recipient layers (L4–L5), whereas lifelong absence of retinal input predominantly impacts deeper layer (L6) interneurons. These results highlight temporally coordinated and complementary roles of thalamic activity and retinal input in shaping the postnatal laminar distribution and density of cortical interneurons.

## Discussion

This study examines whether intrinsic thalamic activity contributes to the integration, positioning, and density of local interneurons in the dorsolateral geniculate nucleus (dLGN), and how early disruptions of this activity affect the development of cortical interneurons.

### Retinal contributions: structural input rather than early Stage I activity

Our retinal manipulation experiments largely recapitulate established findings (Golding et al., 2014; Charalambakis et al., 2019; Su et al., 2020; Somaiya et al., 2022), providing a solid framework for interpreting thalamic contributions. Selective blockade of Stage I retinal waves with carbenoxolone between E18.5–P0 and P0–P1 did not affect interneuron density or spatial distribution in the dLG. In contrast, embryonic bilateral enucleation reduced interneuron number and altered their laminar positioning. Together with previous studies in Math5^−^/^−^ mice and RGC-specific TeNT models, in which blocking glutamate release from P0 onward does not alter interneuron numbers (Su et al., 2020; Somaiya et al., 2022), our data reinforce the idea that retinal axons are necessary for normal interneuron recruitment, whereas Stage I retinal activity is not. A plausible mechanism involves Shh signaling from retinogeniculate axons, which induces astrocytic FGF15 expression and promotes interneuron recruitment (Su et al., 2020; Somaiya et al., 2022). Thus, retinal structural input provides essential molecular cues, but early retinal waves are not sufficient to explain interneuron density regulation.

### Intrinsic thalamic activity as a regulatory driver

While prior work has largely focused on retina-derived signals, less attention has been given to how the developing dLG integrates its own intrinsic activity. By employing complementary genetic models that alter thalamic excitability while preserving retinal innervation, we identify intrinsic thalamic activity as an important contributor to interneuron density and distribution. Importantly, the *Th^Kir^* model does not abolish thalamic firing. Rather, Kir2.1 overexpression disrupts the coordination and temporal structure of intrinsic activity, producing a bistable and desynchronized pattern and an increase in asynchronous activity (Antón-Bolaños et al., 2019). Thus, our findings reflect the consequences of altered network dynamics rather than simple activity deprivation. This distinction is critical, as developmental processes often depend on patterned synchrony rather than absolute firing rates.

Both Kir2.1 overexpression models exhibited a reduced dLG area, consistent with previous reports (Moreno-Juan et al., 2023), accompanied by increased interneuron density. However, the underlying dynamics differed. In *SertCre^Kir^* mice, density increases were evident from P6 onward without changes in absolute interneuron number, suggesting a scaling effect secondary to reduced nucleus size. In contrast, *Th^Kir^* animals exhibited early density alterations (from P2) and a transient increase in interneuron number at P6.

As *Th^Kir^* disrupts activity prenatally and persists postnatally, whereas *SertCre^Kir^* acts perinatally and affects multiple visual nodes, these differences may reflect distinct temporal and network-level influences. Specifically, the difference in P6 interneuron numbers between *Th^Kir^* and *SertCre^Kir^* mice likely reflects the later onset of Kir2.1-mediated disruption in *SertCre^Kir^*, which emerges too late to influence the P6 phenotype. Notably, our ex vivo Ca²^+^ imaging data demonstrate altered activity in the dLG of *SertCre^Kir^* mice at P4, confirming that intrinsic thalamic dynamics are perturbed in this model as well. However, given its mixed retinal and thalamic expression, we interpret *SertCre^Kir^* as a complementary model rather than temporally definitive.

### Temporal interpretation and postnatal contributions

Intrinsic thalamic wave-like events diminish around birth, whereas interneuron number differences in *Th^Kir^* mice become significant at P6. This temporal separation indicates that the phenotype cannot be attributed exclusively to an embryonic wave time window. Instead, we propose that sustained disruption of intrinsic thalamic dynamics across early development influences interneuron positioning and integration. The altered bistable activity pattern and increased frequency of asynchronous activity persist beyond embryonic stages and may influence activity-dependent processes such as synaptic stabilization, competition, or homeostatic scaling during the first postnatal week.

Our cleaved caspase-3 analysis suggests a trend toward increased cell death in noninterneuron populations, which could contribute to the overall reduction in dLG size observed in this model. However, the absence of increased cell death in interneurons indicates that the transient P6 increase in interneuron number in *Th^Kir^* mice is not explained by preferential apoptosis. Rather than local cell death, we consider the possibility of spatial redistribution, with interneurons relocating to ectopic regions outside the nucleus, as previously observed in Math5^−^/^−^ mice (Su et al., 2020). Thus, intrinsic thalamic activity may regulate spatial retention and allocation within the nucleus. Importantly, our data support a broader conclusion: intrinsic thalamic activity across developmental stages regulates interneuron density in the developing visual thalamus.

Our findings argue against viewing the dLG as a passive relay of retinal signals. Instead, the developing thalamus emerges as an active integrative hub where sensory input and intrinsic network dynamics converge to shape inhibitory circuit assembly. Astrocytes may play a central role in this process. Beyond mediating Shh-dependent recruitment signals, astrocytes are well positioned to sense global activity levels and translate them into molecular cues that regulate interneuron incorporation. We speculate that intrinsic thalamic activity may engage astrocyte-dependent homeostatic mechanisms that adjust interneuron recruitment or positioning according to network demands. While this hypothesis requires direct testing, it offers a framework linking activity patterns to structural circuit assembly.

### Consequences for cortical interneuron development

Disruption of intrinsic thalamic activity also impacts cortical interneuron development in V1. In *Th^Kir^* mice, SST^+^ interneurons displayed a transient increase in density in L4 at P6 and P15, normalizing by P30. This may reflect compensatory adjustments to altered cortical excitability previously described in this model (Antón-Bolaños et al., 2019), consistent with the role of SST^+^ interneurons in regulating early cortical activity (Marques-Smith et al., 2016; Modol et al., 2024).

In contrast, PV^+^ interneurons were increased in L4 and L5 at P30, suggesting altered maturation trajectories or reduced programmed cell death. Since SST^+^ interneurons influence PV^+^ maturation (Xu et al., 2013; Tuncdemir et al., 2016; Naka et al., 2019), early SST^+^ overrepresentation may contribute indirectly to delayed PV^+^ refinement. Alternatively, persistent disruption of thalamocortical input (Antón-Bolaños et al., 2019; Duan et al., 2020) may directly influence PV^+^ development.

Despite laminar shifts, total GAD67^+^ interneuron density remained largely stable, with only a transient increase at P15. This suggests subtype-specific adjustments rather than global overproduction, possibly reflecting activity-dependent compensatory mechanisms during the transition to sensory-driven activity after eye opening (Anastasiades et al., 2016; Wong et al., 2018).

In the embBE model, permanent removal of retinal input also altered cortical interneuron localization, particularly in L6. SST^+^ interneurons exhibited a biphasic redistribution, while PV^+^ interneurons were increased at later stages. These findings suggest that retinal input contributes to proper laminar allocation and maturation timing of cortical interneurons. Interestingly, PV^+^ interneurons were increased in L4 at P30 in both embBE and *Th^Kir^* mice, which may reflect delayed maturation or prolonged circuit immaturity, potentially influencing critical period timing (Fagiolini et al., 2004; Hensch, 2005).

### Conclusion

Together, our findings demonstrate that both retinal and thalamic components of the visual pathway contribute to interneuron development at multiple hierarchical levels. Rather than acting independently, retinal structural cues and intrinsic thalamic activity interact to shape interneuron recruitment, positioning, and cortical integration. The first postnatal week emerges as a particularly sensitive period during which sustained intrinsic thalamic dynamics influence inhibitory circuit assembly. Disruption of these early activity patterns may have long-lasting consequences for excitation–inhibition balance and circuit maturation. Understanding how intrinsic activity and molecular signaling converge to shape interneuron development provides insight into mechanisms by which early network perturbations could predispose to neurodevelopmental disorders.
